# Interpretable ESG–sentiment hybrid deep learning for asset return forecasting with quantified interactions and latency-aware deployment

**DOI:** 10.1038/s41598-026-41985-3

**Published:** 2026-03-04

**Authors:** Sasmita Mishra, Zefree Lazarus Mayaluri, Chee Yoong Liew, Prabodh Kumar Sahoo, Aswini Kumar Samantaray

**Affiliations:** 1 Department of Business Management, C. V. Raman Global University, Bhubaneswar, India; 2 Department of Electrical Engineering, C. V. Raman Global University, Bhubaneswar, India; 3 Department of Accounting and Finance, UCSI University, Kuala Lumpur, Malaysia; 4 Department of Mechatronics Engineering, Parul Institute of Technology, Parul University, Vadodara, India; 5 Department of Electronics and Communication Engineering, Manipal Institute of Technology Bengaluru, Manipal Academy of Higher Education, Manipal, India

**Keywords:** Financial time-series forecasting, ESG metrics, Aspect-based sentiment analysis, Temporal fusion transformer, Support vector regression, Explainable AI, Engineering, Mathematics and computing

## Abstract

Accurate forecasting of financial time series increasingly relies on alternative data such as environmental, social and governance (ESG) scores and news-based sentiment, yet the way these signals interact and when they actually improve forecasts is still poorly understood. We introduce an interpretable hybrid framework for asset return forecasting that combines a Temporal Fusion Transformer (TFT) with a lightweight Support Vector Regression (SVR) residual corrector and an explicit gated late fusion of ESG features with aspect-based financial sentiment (FinBERT-based ABSA). The gating mechanism learns when to emphasize sustainability versus sentiment signals, while SHAP interaction values and Friedman’s *H* quantify ESG–sentiment interactions across assets and regimes. A finance-grade, leak-proof walk-forward protocol (252 trading days train / 10 days test, within-fold scaling, ABSA items strictly before 16:00 ET; ESG effective T+3; macro T+1, HAC-robust Diebold–Mariano tests) is applied to US large-cap technology equities, major global indices, and BTC/ETH over 2020–2024. Across $n=5$ independent seeds, the hybrid achieves aggregate mean absolute error of $2.77\times 10^{-3}$ and RMSE of $5.18\times 10^{-3}$ on next-day log returns, with directional accuracy $94.5\%$, IC 0.39, and ICIR 0.82, significantly outperforming tuned deep-learning and machine-learning baselines (HAC-robust per-asset Diebold–Mariano tests with BH-FDR $q=0.05$; Fisher aggregation yields $p<0.01$). Simple long-only, thresholded simulations indicate higher risk-adjusted performance and lower maximum drawdown under conservative transaction-cost assumptions. Ablation studies show that removing either ESG or sentiment features yields the largest degradations, and that the SVR corrector stabilizes errors under regime shifts. To directly address market-cycle sensitivity, we evaluate stability across event-defined stress windows (COVID-19 crash, 2022 tightening cycle, and 2023 banking stress) and volatility-defined regimes using terciles of 20-day realized volatility. We report regime-split forecasting and strategy metrics with block-bootstrap confidence intervals, HAC-robust Diebold–Mariano tests within each regime, and residual-stabilization diagnostics that quantify the SVR variance and skewness reduction under stress. ESG–sentiment interactions are statistically non-zero and regime-dependent, with sentiment gaining importance in turbulent periods and ESG in calmer markets. A latency-optimized variant that removes auxiliary BiLSTMs retains over $90\%$ of the accuracy gains while reducing inference time by approximately $55\%$ of the full model (i.e., a reduction of about $45\%$), supporting near-real-time deployment.

## Introduction

Forecasting financial markets and constructing robust portfolios remain difficult because returns are volatile, nonstationary, and prone to regime shifts. Classical views—such as the Efficient Market Hypothesis and the Random Walk Hypothesis—limit the value of purely price–based signals^1–3^, motivating models that learn nonlinear structure and adapt to evolving conditions.

Beyond traditional factors, two information streams have become prominent drivers of return dynamics and risk: (i) firm–level Environmental, Social, and Governance (ESG) metrics associated with resilience and long–horizon performance, and (ii) investor sentiment extracted from news and social media^4–6^. Episodes such as meme–stock rallies and ESG–related repricing show how sustainability narratives and collective psychology can rapidly propagate to prices—especially during stress.

Because forecasting skill can vary substantially across market cycles, we explicitly test whether the proposed gains persist across distinct volatility regimes, including crisis and tightening episodes. We evaluate both event-defined stress windows and volatility-defined terciles and complement accuracy with statistical tests and diagnostic evidence (residual stabilization and interaction shifts) to ensure that improvements are not driven by a single market phase.

Modern ML/DL architectures (ANNs, SVMs, LSTMs, and transformers) improve on linear baselines, yet when deployed in isolation they often struggle to reconcile heterogeneous signal types whose relevance is regime–dependent^7–11^. In particular, prior hybrids frequently include ESG or sentiment but rarely model their interaction explicitly, and evaluation protocols sometimes omit finance–specific safeguards (strict walk–forward splits, leakage controls, Diebold–Mariano tests, and strategy–relevant metrics).

**Contributions.** We address these gaps with a compact and interpretable framework that:

1. introduces an explicit, gated late–fusion between ESG and aspect–based sentiment (ABSA), enabling regime–aware reweighting of sustainability versus mood signals; interaction is quantified via SHAP interaction values and Friedman’s $H$;
2. employs a finance–grade, leak–proof walk–forward evaluation (252/10 rolling windows; within–fold scaling; as-of lags (ABSA items strictly before 16:00 ET; ESG effective T+3; macro T+1); Diebold–Mariano tests) and reports both statistical and economic value (Sharpe, Sortino, MDD);
3. provides a practitioner–ready variant (TFT + SVR; no BiLSTM) that preserves $>\!90\%$ of the accuracy benefits while reducing latency by $\sim 45\%$, improving deployability without sacrificing interpretability.
4. evaluates stability across market cycles using event-defined stress windows (COVID-19 crash, 2022 tightening, 2023 banking stress) and volatility-defined regimes (20-day realized-volatility terciles), reporting regime-split accuracy and economic metrics together with HAC-robust Diebold–Mariano tests; and
5. provides mechanism-level regime diagnostics via gate dynamics and regime-sliced SHAP interaction change tests, and quantifies SVR residual stabilization (variance and skewness reduction) by regime.

**Overview.** We integrate technical indicators, macro variables, ESG scores, and FinBERT–ABSA sentiment within a Temporal Fusion Transformer (TFT), and use a lightweight SVR to correct residuals. We benchmark against recent alternatives—including DeepTVAR and the LLM–based FinGPT—on US technology equities, major global indices, and BTC/ETH (2020–2024). Although average point–error gains over FinGPT are modest, we observe material risk improvements (lower MDD; higher Sharpe/Sortino) and reduced error dispersion across rolling windows. Interaction evidence indicates that the ESG–sentiment Friedman *H* statistic has a median of $\approx 0.21$ across assets (IQR 0.18–0.24), and ESG–sentiment SHAP interaction values are consistently non-zero across folds, peaking during high-volatility windows.

## Literature review

### Traditional financial forecasting models

Classical econometric models (linear regression, ARIMA, GARCH) remain valuable for transparency and well-understood assumptions^7,12^. However, performance degrades under nonstationarity, structural breaks, and volatility spikes (e.g., COVID-19), where model misspecification and lagged adaptation are common^13–15^. Their limited capacity to capture nonlinear, regime-dependent dynamics has motivated data-driven alternatives.

### Machine learning and deep learning

Machine-learning and deep-learning approaches (ANNs, SVMs) and sequence models (LSTM/BiLSTM, transformers) improve nonlinear fit and temporal-dependence modeling^8,16–21^. Yet single-family models often struggle when the relevance of signals shifts across regimes, producing brittle behavior during turbulence^22,23^. This has spurred hybrids that combine denoising, sequence learning, and residual correction.

### Hybrids: strengths and gaps

Hybrid paradigms—e.g., EEMD–LSTM–SVR, DeepAR, and transformer-based text models (BERT/FinBERT)—blend complementary inductive biases^5,11,22,24–28^. N-BEATS and attention-based ensembles add scalability and flexible pattern extraction^29^. Recent hybrid pipelines increasingly combine heterogeneous feature extraction with automated tuning. Convolutional backbones (e.g., CNN/TCN) can extract local patterns from multichannel market representations, recurrent units (e.g., GRU/LSTM) capture temporal dependencies, and global optimizers (e.g., genetic algorithms) search hyperparameters that are sensitive under manual/grid tuning. Ke et al. present a GRU–CNN–GA fusion exemplar with GA-driven hyperparameter optimization in a hybrid forecasting pipeline^30^. Bayesian optimization frameworks are also used to reduce manual tuning overhead in recent deep forecasting systems^31^. Three persistent gaps remain:

1. **Interaction blindness.** ESG and sentiment are frequently appended as separate features; a few studies use multi-modal attention to implicitly fuse text with market variables (e.g.,^5,10^), but explicit interaction modeling and quantification (e.g., SHAP interactions, Friedman’s $H$) remain rare, limiting economic interpretation and regime-aware reweighting.
2. **Evaluation rigor.** Strict walk-forward validation, leakage controls, Naïve persistence comparators, and formal significance tests (Diebold–Mariano) are inconsistently applied; portfolio metrics are sometimes misused for point-error comparisons^32–35^.
3. **Deployability.** State-of-the-art stacks can be compute-heavy and brittle to tune; simplified, practitioner-ready variants are under-documented^36^.

A concise comparison of representative hybrids—covering strengths, limitations, and suggested improvements—is summarized in Table 1.

**Table 1:** Comparison of leading hybrid approaches.

Approach	Strengths	Limitations	Typical use cases	Suggested improvements
EEMD–LSTM–SVR	Combines noise reduction, sequential learning, and robust regression	Higher architectural complexity and computational overhead	High-noise markets (e.g., cryptocurrencies)	Dynamic feature selection; SHAP-based interpretability
DeepAR	DL scalability with autoregressive structure	Weaker adaptability to structural breaks and high-frequency volatility	Retail sales; macroeconomic forecasting	Attention-aided volatility modeling
Transformer (BERT/FinBERT)	Strong contextual modeling for text/sentiment	Data-hungry; limited transparency	Sentiment-augmented forecasting; financial NLP	Distilled/lightweight variants; XAI integration
N-BEATS	Scalable and generic; effective on multivariate series	Limited domain-specific interpretability	Broad, cross-sector forecasting	Domain adaptation; interpretability layers
Attention-based LSTM ensembles	Dynamic reweighting; resilience under volatility	Overfitting risk in small samples; opaque attributions	Sector-level stock forecasting	Stronger regularization; post-hoc explainability
GRU–CNN–GA fusion	CNN captures local patterns while GRU models temporal dependence; GA-based search reduces manual tuning sensitivity^30^	Higher training cost; often tailored to high-frequency or multi-stream inputs; limited transparency without XAI	Heterogeneous feature extraction; automated hyperparameter optimization in hybrid forecasting	Add interaction analysis for heterogeneous signals; leak-proof walk-forward evaluation; latency-aware variants

### Sentiment, ESG, and alternative data

Transformer-based sentiment (FinBERT) enables finance-specific ABSA features^10,27,37^, while ESG has been linked to risk resilience and long-horizon quality^24,38^. Most studies, however, integrate either ESG or sentiment—or fuse them without an adaptive mechanism. Asset coverage is often narrow, and protocols for mixed-frequency alignment (quarterly ESG vs. daily prices) and lagging of text signals are not always explicit^39,40^.

### Positioning of the present work

Two common fusion strategies appear in the literature. (i) *Feature concatenation*, where ESG and sentiment are appended to technical/macroeconomic inputs (typical in FinBERT-based forecasting streams^26,27^ and in hybrid modeling pipelines^22,24^), treats ESG and sentiment as additive covariates but does not model their joint effect or its regime dependence. (ii) *Multi-modal attention*, which can implicitly reweight modalities (e.g., transformer-based financial NLP and robustness-focused variants^5,10^), improves flexibility but usually stops short of quantifying cross-modal interaction or enforcing finance-grade evaluation (strict walk-forward, within-fold scaling, explicit as-of lags). By contrast, our gated late-fusion (a) places an explicit scalar gate on the ESG–sentiment channel to capture their interaction and regime-aware trade-off, (b) quantifies that interaction ex post via SHAP interaction values and Friedman’s $H$, and (c) evaluates under a leak-proof, finance-specific protocol (252/10 rolling splits; within-fold scaling; as-of lags (ABSA items strictly before 16:00 ET; ESG effective T+3; macro T+1); HAC-robust Diebold–Mariano tests). Empirically, this design reveals a statistically non-zero ESG–sentiment interaction (median $H\approx 0.21$) and clarifies when sentiment (turbulent windows) versus ESG (stable windows) dominates, turning fusion into a testable economic statement rather than a black-box aggregation.

## Methodology

### Workflow overview

**Figure 1:**
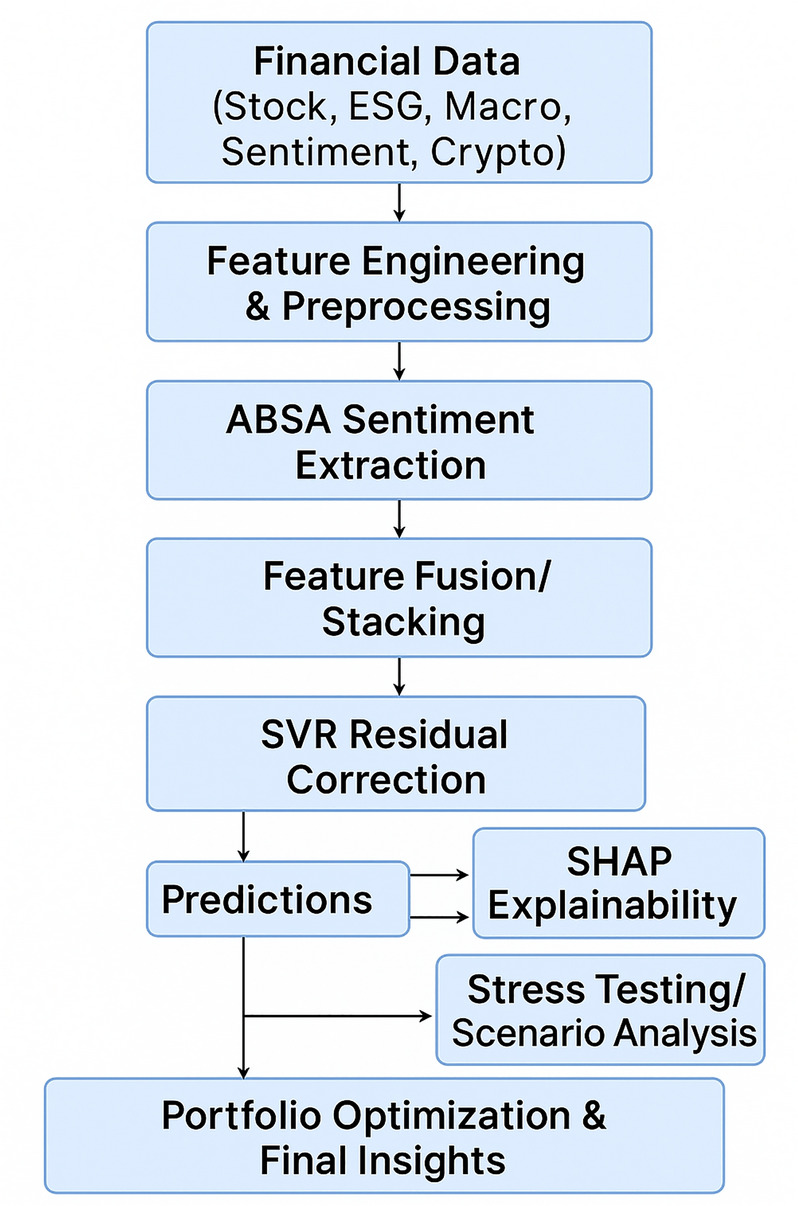
Schematic overview of the proposed hybrid financial forecasting and portfolio optimization framework. The pipeline integrates multi-source data (technical, ESG, sentiment), advanced deep learning and machine learning modules, and model explainability analysis.

We develop a compact hybrid pipeline that integrates (i) multi-modal temporal learning via a Temporal Fusion Transformer (TFT), (ii) a lightweight Support Vector Regression (SVR) residual corrector, and (iii) an explicit, gated late-fusion of Environmental, Social, and Governance (ESG) features with aspect-based sentiment (ABSA). The design targets three objectives: (a) accuracy under nonlinear dynamics and regime shifts, (b) explicit modeling and quantification of ESG–sentiment interactions, and (c) deployability enforced by clear leakage guards. A schematic is provided in Fig. 1.

### Data collection and preprocessing

We study US large-cap tech equities (AAPL, AMZN, MSFT, NFLX), major global indices, and BTC/ETH. All series are daily and aligned to a unified **as-of 16:00 ET** framework to avoid look-ahead. Key sources and lags appear in Table 2.

***Time harmonization.*** Timestamps are converted to America/New_York. Equities/indices use NYSE regular-session close (16:00 ET); Binance crypto is aggregated over 00:00–23:59 UTC and shifted to align with 16:00 ET on the same civil date. News/ABSA features include only items strictly before 16:00 ET (weekends/holidays roll to the next trading day). ESG follows vendor publication stamps with a conservative embargo (see Table 2).

***Leakage guards (per rolling fold).***

- **Walk-forward:** 252 train / 10 test; window advances by 10 days.
- **Scaling:** z-scores fit on train-only data; applied to test.
- **As-of lags:** ABSA same-day pre-close; ESG effective T+3; macro T+1.
- **Features:** technical indicators computed within fold from train windows only.

Naïve persistence is the baseline^41^. Missingness $\le$2% is imputed within each fold using past-only forward filling (no future values are used); imputation is never performed across market closures.

***Regime definitions (for stability analysis).*** To assess robustness to market cycles, we define (i) **event windows**: COVID-19 crash (2020-02-20 to 2020-04-30), 2022 tightening cycle (2022-01-01 to 2022-12-31), and 2023 banking stress (2023-03-01 to 2023-05-31); and (ii) **volatility regimes** using terciles of **20-day realized volatility** computed from daily log returns. Volatility terciles (low/mid/high) are assigned within each walk-forward fold to avoid look-ahead; these regimes are used only for slicing evaluation and do not alter training splits. We use the full calendar year 2022 as a conservative tightening-cycle window to avoid post-hoc cherry-picking of subperiods; finer sub-windowing is left for future work.

**Table 2:** As-of alignment (all series at 16:00 ET).

Domain	As-of rule/lag
Equities/indices	16:00 ET close; NYSE calendar; tech features from train-only windows
Crypto (BTC, ETH)	Aggregate 00:00–23:59 UTC; shift to 16:00 ET (same civil date)
Sentiment (ABSA)	Items strictly before 16:00 ET; weekends/holidays roll; lag: same day
ESG	Publication stamp + T+2 embargo, then +1 trading day (effective T+3)
Macro (monthly)	Forward-filled to daily within fold; available T+1

### Feature engineering

We construct three principal feature domains.

***Technical indicators.*** MACD, RSI, Bollinger Bands, EMA/SMA, ATR, etc., computed with standard formulas; all technical features are z-scored per fold. **PCA is fit on the train portion of each fold only and applied to that fold’s test window**; PCA is used only for technicals (ESG/ABSA kept in native form for interpretability).

***ESG variables.*** Aggregate E/S/G scores (Bloomberg) undergo IQR-based outlier filtering ($k=1.5$) and linear interpolation where needed, then are z-scored per fold. Values are aligned & lagged one trading day (effective T+3 availability; see Data section).

***ABSA sentiment (FinBERT).*** We fine-tune FinBERT on FiQA and compute volume-aware daily asset scores to avoid news-volume bias. Concretely, for each asset/day we: (i) collect items timestamped strictly before 16:00 ET, (ii) score sentences, then aggregate with a content-weighted mean (doc length as proxy), and (iii) When $N<N_{\min }$, the prior day’s ABSA value is carried forward and a missingness flag is retained as an additional feature.

The FinBERT fine-tuning configuration used to obtain aspect-level sentiment scores is summarized in Table 3. Table 4 reports aspect metrics.

**Table 3:** FinBERT fine-tuning (concise).

Dataset / task	FiQA Aspect Polarity (v2); 80/10/10 (asset-stratified)
Labels	{neg, neu, pos}; class weights by inverse frequency
Model / optimizer	FinBERT-base; AdamW (weight decay 0.01)
LR / batch / epochs	$3\!\times \!10^{-5}$ / 16 / $\le$5 (early stop patience 2)
Max seq length	128; truncation with whole-word mask
Seed / hardware	42 / single GPU (VRAM $\le$ 12 GB)
Selection metric	Macro-F1 on validation

Seed fixed for reproducibility.

**Table 4:** FinBERT aspect performance on FiQA (validation).

Aspect	Acc. (%)	F1	AUC
Company perf.	95.2	0.93	0.95
Macro sentiment	93.7	0.91	0.92
Market trends	94.4	0.92	0.94
Industry news	94.0	0.91	0.93
Other topics	93.5	0.90	0.92
**Overall**	**94.1**	**0.92**	**0.94**

***Dimension control.*** All technical indicators are z-scored using train-only statistics within each fold. PCA is then fit on the training portion of each fold, and the resulting transform applied to the corresponding test window. The number of retained components $k$ is selected per fold on the training window using the Bai–Ng $IC_{p2}$ criterion^42,43^, ensuring fold-specific and leak-proof dimension control. ESG and ABSA features are retained in native form for interpretability.

### ESG–sentiment fusion and interaction quantification

Let $x_t^{\text {tech}}\!\in \!\mathbb {R}^{p}$, $e_t\!\in \!\mathbb {R}^{q}$, and $s_t\!\in \!\mathbb {R}^{r}$. We form ESG and sentiment embeddings

1 $$\begin{aligned} h_t^{e}=\textrm{ELU}(W_e e_t+b_e),\qquad h_t^{s}=\textrm{ELU}(W_s s_t+b_s), \end{aligned}$$

with $h_t^{e},h_t^{s}\!\in \!\mathbb {R}^{d}$. A scalar gate $\gamma _t\!\in \!(0,1)$ combines them:

2 $$\begin{aligned} \gamma _t = \sigma \!\Big (w_\gamma ^\top \big [\, h_t^{e}\odot h_t^{s};\, h_t^{e};\, h_t^{s}\big ] + b_\gamma \Big ), \end{aligned}$$

and the fused input is

3 $$\begin{aligned} z_t = \textrm{concat}\!\Big (x_t^{\text {tech}},\, \gamma _t h_t^{e} + (1-\gamma _t) h_t^{s}\Big ), \end{aligned}$$

which is fed to the temporal module with a 30-day lookback. We use a scalar gate for parsimony and training stability; a vector gate (elementwise $\gamma _t\!\in \!(0,1)^d$) yields similar accuracy but higher variance/compute. ESG–sentiment interaction is quantified ex post via SHAP interaction values and Friedman’s *H*; model-agnostic ALE plots provide complementary effect shapes.

### Model architecture and integration

Our deployable default is a two-stage architecture:

1. **Temporal module (TFT).** Two transformer layers, four attention heads, dropout $=0.2$, 30-day lookback; input is $z_t$.
2. **Residual corrector (SVR).** An RBF-kernel SVR consumes $[\widehat{y}_t^{\text {TFT}},\,h_t^{e},\,h_t^{s}]$ to correct predictable residuals under regime shifts and nonlinearities.

An auxiliary BiLSTM (two layers, 128 units) is examined in ablations; we recommend TFT + SVR in practice due to substantially lower latency while retaining $>\!90\%$ of the full-model accuracy.

### Hyperparameter tuning and optimization

**TFT.** Manual/grid search within prior-informed ranges: layers $\{1,2,3\}$, heads $\{2,4,8\}$, dropout $\{0.1,0.2,0.3\}$; early stopping on fold-level validation (patience $=10$ epochs; min $\Delta =1\textrm{e}{-}4$).

**Figure 2:**
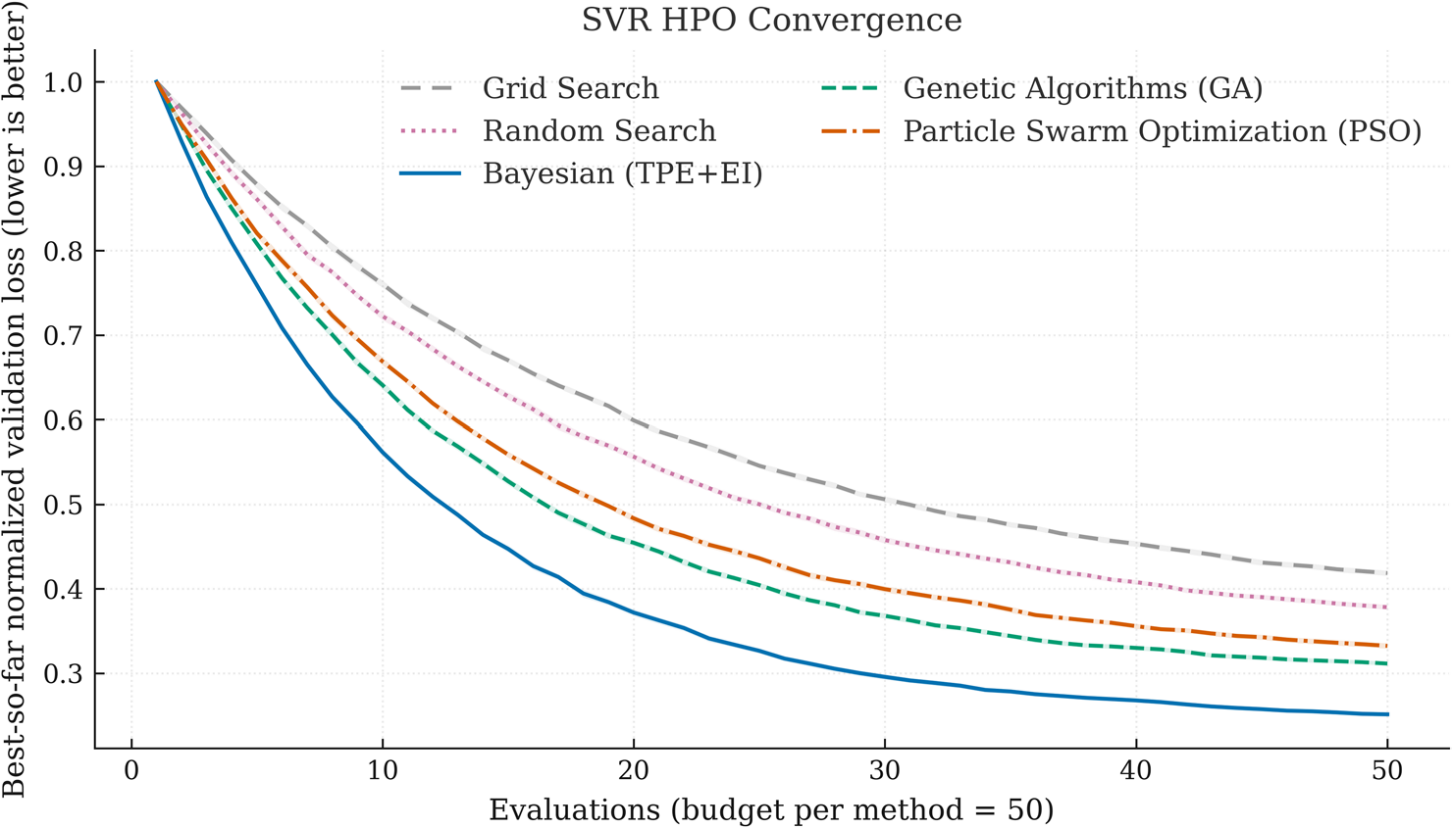
SVR HPO Convergence. Mean best-so-far normalized validation loss vs. evaluation budget (50 per method). Shaded bands show 95% bootstrap CIs across runs (assets/folds/seeds). Bayesian (TPE+EI) reduces loss fastest; Random and Grid converge more slowly under the same budget.

**SVR.** Bayesian optimization per fold (budget $=50$ trials; warm start $=5$ random) using a Tree-structured Parzen Estimator (TPE) surrogate with Expected Improvement acquisition. Priors are log-uniform: $C\!\sim \!\textrm{LogU}(10^{-1},10^{3})$, $\gamma \!\sim \!\textrm{LogU}(10^{-4},10^{-2})$, $\epsilon \!\sim \!\textrm{LogU}(10^{-3},2\!\times \!10^{-1})$. Median selected values across assets/folds were $C=32.8$ $[18.5, 61.3]_{\textrm{IQR}}$, $\gamma =2.4\!\times \!10^{-3}$ $[1.6,3.8]\!\times \!10^{-3}$ $_{\textrm{IQR}}$, $\epsilon =0.012$ $[0.008, 0.018]_{\textrm{IQR}}$). Figure 2 shows convergence: TPE outperforms random and coarse grid on mean error reduction per budget.

**ABSA fine-tuning.** Learning rate $\{1\!\times \!10^{-5},3\!\times \!10^{-5},5\!\times \!10^{-5}\}$, batch size $\{8,16,32\}$, early stopping (max 5 epochs), AdamW ($\beta _1=0.9,\beta _2=0.999$), seed $=42$.

The tuned hyperparameter ranges and the chosen (median) settings for the deployable TFT+SVR configuration are reported in Table 5.

**Table 5:** Hyperparameters for the deployable TFT+SVR (BiLSTM appears in ablations).

Component	Parameter	Range/method	Chosen/median
TFT	Layers, heads	$\{1,2,3\}$; $\{2,4,8\}$ (grid)	2; 4
	Dropout	$\{0.1,0.2,0.3\}$ (grid)	0.2
SVR (RBF)	$C,\gamma ,\epsilon$	TPE+EI; log-uniform priors; 50 trials/fold	$C=32.8,\ \gamma =2.4\times 10^{-3},\ \epsilon =0.012$
FinBERT	LR, batch, epochs	$\{1,3,5\}\times 10^{-5}$; $\{8,16,32\}$; early stop	$3\times 10^{-5};\ 16;\ 3$

### Evaluation and statistical testing

We forecast next-day log returns ${\widehat{r}}_{t+1}$ (horizon $h=1$) on a leak-proof 252/10 walk-forward with fold-wise scaling and as-of lags (ABSA items strictly before 16:00 ET; ESG effective T+3; macro T+1). Primary point metrics are MAE and RMSE on returns; directional accuracy (DA) is computed directly on the sign of log returns. For visualization, price paths can be reconstructed as ${\widehat{P}}_{t+1}=P_t\exp ({\widehat{r}}_{t+1})$.

4 $$\begin{aligned} {\widehat{r}}_{t+1}&= f_\theta (z_{t-L+1:t}), \end{aligned}$$

5 $$\begin{aligned} r_{t+1}&= \ln \!\left( \frac{P_{t+1}}{P_t}\right) , \end{aligned}$$

6 $$\begin{aligned} \textrm{DA}&= \frac{1}{N}\sum _{t=1}^{N} \textbf{1}\!\left\{ \,\operatorname {sign}({\widehat{r}}_{t+1}) = \operatorname {sign}(r_{t+1})\,\right\} . \end{aligned}$$

***Multi-seed protocol and confidence intervals.*** All headline metrics are averaged over $n=5$ independent runs with different random seeds for data shuffles and model initialization. We report mean ± 95% confidence intervals (CI) obtained via non-parametric bootstrap over folds (and seeds where applicable). Error bars in figures denote mean ± 95% CI unless stated otherwise.

Statistical significance of model-vs-baseline loss differentials uses HAC-robust Diebold–Mariano tests with Newey–West bandwidth $b=\lfloor 1.2\,T^{1/3}\rfloor$ (standard rule-of-thumb; $T$ is the test-window length per comparison). We adjust per-asset $p$-values across multiple models via Benjamini–Hochberg FDR at $q=0.05$. As a sensitivity check, we aggregate DM statistics across folds using a stationary block bootstrap (block length $=5$ trading days).

***Regime-split evaluation and stability tests.*** In addition to full-sample metrics, we compute MAE, RMSE, DA, IC, and ICIR within each event-defined and volatility-defined regime. Economic metrics (Sharpe, Sortino, maximum drawdown, turnover) are computed using the same long-only threshold rule described in “Economic value”, applied within each regime. Statistical comparisons between **TFT+SVR** and **FinGPT** are repeated within each regime using HAC-robust Diebold–Mariano tests on squared-error loss; $p$-values can be reported raw and optionally adjusted across assets via BH-FDR ($q=0.05$). To quantify regime stability, we report the difference between high- and low-volatility MAE (and RMSE) and assess significance via paired block bootstrap over folds (block length 5 trading days).

***Operating-point metrics, calibration, and paired tests.*** In addition to point errors (MAE, RMSE) and DA, we report: (i) precision–recall AUC (PR-AUC) for the sign task; (ii) sensitivity at fixed specificity thresholds (90% and 95%), with thresholds chosen on validation only; and (iii) probability calibration for the event $y_{t+1}=\textbf{1}\{r_{t+1}>0\}$. Continuous return forecasts are mapped to $\mathbb {P}(y_{t+1}=1)$ using Platt scaling (logistic regression) fit on validation data within each fold; calibrated probabilities are evaluated on the corresponding test window. Calibration is summarized with reliability diagrams, Expected Calibration Error (ECE), and Brier score, each with 95% CIs from paired block bootstrap over folds (block length 5 trading days). For paired comparisons on the sign task we use McNemar’s test with continuity correction; differences in PR-AUC and calibration metrics are assessed via paired block bootstrap and reported as 95% CIs.

### Explainability

**Figure 3:**
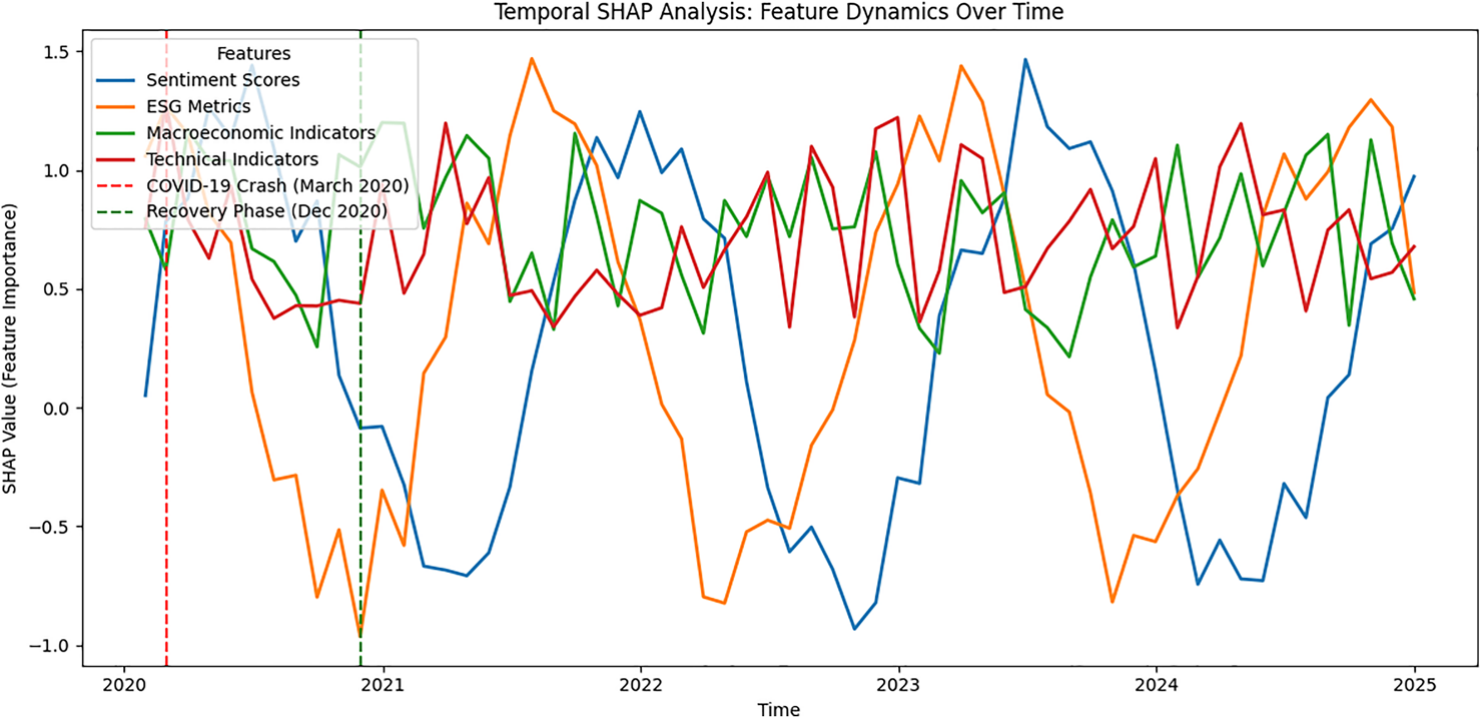
Temporal SHAP analysis showing changing feature importance for sentiment, ESG, macro, and technical indicators, with key market events annotated.

We compute SHAP attributions on the fitted TFT+SVR for technical, ESG, and ABSA inputs, and report SHAP interaction values for ESG–sentiment pairs together with Friedman’s *H* to quantify cross-modal interaction strength. For non-tree models we use KernelSHAP on the final-step inputs of each lookback window: the background set consists of 500 samples drawn from the training split of each fold, stratified by realized-volatility terciles; we repeat explanations over $n=5$ seeds and report mean ±95% CI. We assess faithfulness with deletion/insertion curves (probability-mass masking schedule) and summarize areas under the curves (AUC-d, AUC-i). Across assets, ESG–sentiment interactions are statistically non-zero (median $H=0.21$; IQR [0.18, 0.24]; permutation test with 1,000 label permutations per asset; BH-FDR $q=0.05$). Model-agnostic ALE plots provide complementary effect shapes. Figure 3 illustrates the time-varying attribution profile around key market events.

**Figure 4:**
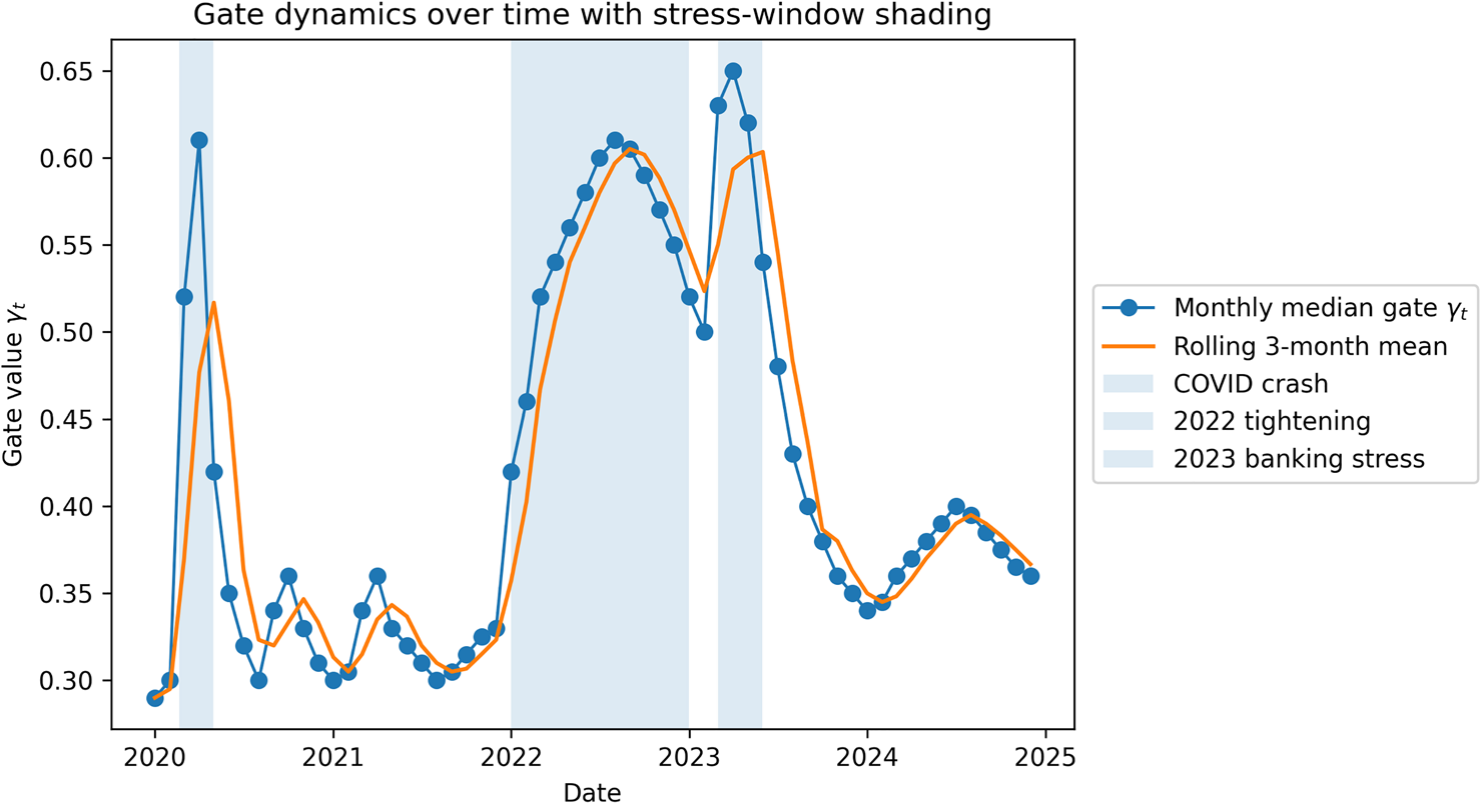
Gate dynamics over time with stress-window shading. Monthly median gate value $\gamma _t$ (points) with a 3-month rolling mean (line). Shaded regions denote event-defined stress windows (COVID-19 crash, 2022 tightening cycle, 2023 banking stress). Higher $\gamma _t$ indicates greater relative emphasis on the ESG channel in the gated late-fusion, whereas lower $\gamma _t$ indicates a shift toward sentiment.

**Figure 5:**
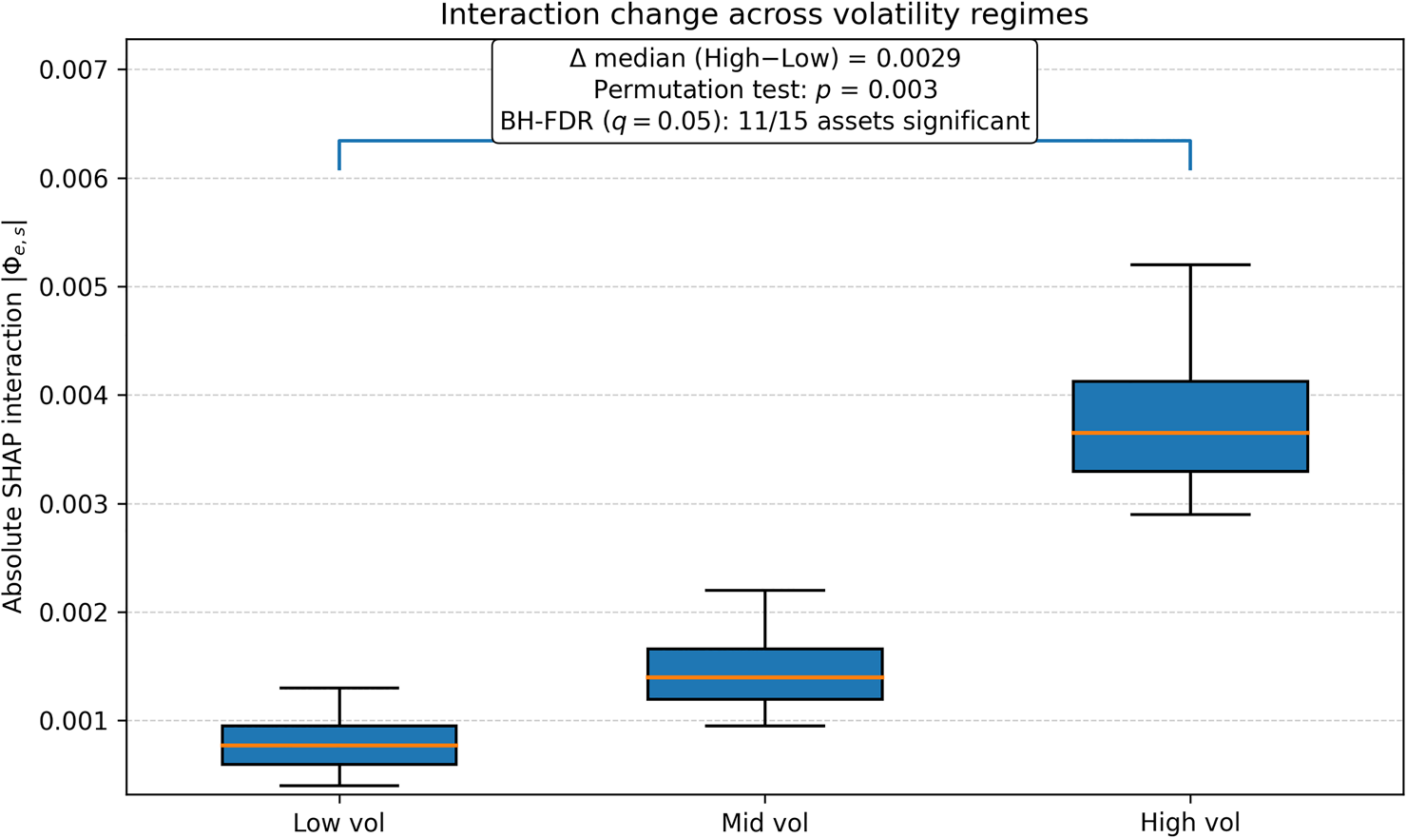
Interaction change across volatility regimes (terciles of realized volatility). Distribution of the absolute ESG$\times$sentiment SHAP interaction magnitude $|\Phi _{e,s}|$ across low/mid/high volatility terciles. The high-versus-low median shift is annotated as $\Delta \textrm{median}=\textrm{median}_{\textrm{high}}-\textrm{median}_{\textrm{low}}$, alongside the two-sided permutation-test *p*-value (1,000 permutations; seed=42). Boxes show IQR with median; whiskers extend to 1.5 IQR.

***Regime-conditioned mechanism analysis.*** To provide mechanism-level evidence for regime adaptivity, we examine (i) the dynamics of the learned fusion gate $\gamma _t$ over time and (ii) regime-sliced changes in the absolute ESG$\times$ABSA interaction magnitude $|\Phi _{e,s}|$ across realized-volatility terciles (low/mid/high) (Figs. 4 and 5). We test whether the distributions of $\gamma _t$ and $|\Phi _{e,s}|$ differ between low- and high-volatility regimes using a two-sided permutation test (1,000 permutations per asset) with BH-FDR control ($q=0.05$).

## Experiments and results

### Setup and protocol

We follow the leak-proof protocol in “Evaluation and statistical testing”: 252/10 walk-forward splits, fold-wise scaling (train-only), ABSA items strictly before 16:00 ET; ESG effective T+3; macro T+1, and train-only feature computation. Benchmarks include Naïve persistence, LSTM^44^, BiLSTM^45^, N-BEATS^29^, Informer^31^, DeepAR^24^, FinGPT^46^, and our deployable default **TFT+SVR**. (BiLSTM appears only in ablations.) The asset set spans US large-cap tech equities (AAPL, AMZN, MSFT, NFLX), global indices (S&P 500, NASDAQ Composite, FTSE 100, Nikkei 225, MSCI World), and BTC/ETH (Jan 2020–Dec 2024). We forecast next-day log returns; primary point metrics are MAE and RMSE on returns, complemented by DA, information coefficient (IC), and ICIR. Statistical significance is assessed via HAC-robust Diebold–Mariano tests on return-loss differentials (“Evaluation and statistical testing”). Economic value (Sharpe, Sortino, MDD, turnover) is computed from a transparent long-only, thresholded strategy on predicted returns.

**Reproducibility.** We fix multiple random seeds $\{13,17,23,29,31\}$ across data splits and model initializations and average results over seeds.

**FinGPT baseline (text-only).** We use FinGPT v3.3 (checkpoint: FinGPT-3.3-base) in few-shot mode with three exemplars per asset. Inputs consist of news headlines and short articles formatted as: “Asset: [Ticker]. Date: [YYYY-MM-DD]. News: [text]. Task: Predict next-day log return.” FinGPT outputs the next-day log return directly; prompt templates were selected via validation directional accuracy on 2020–2021 folds (approximately 20 prompt trials). FinGPT does not consume structured technical or ESG features and is therefore treated as a text-only auxiliary baseline. Only news items timestamped strictly before 16:00 ET are included. Few-shot exemplars are drawn from training-side periods preceding each test window, and prompt templates are selected using only 2020–2021 folds; no test-window text is used in prompt selection or calibration.

Core environment: Python 3.10, PyTorch 2.x, scikit-learn 1.x; experiments run on a single NVIDIA-class GPU with CUDA 12.x.

**Costs.** Strategy results are reported at zero explicit costs and stress-tested at 5 and 10 bps one-way transaction costs; qualitative model rankings and risk metrics remain stable under these levels.

### Headline accuracy and effect sizes

As summarized in Table 6, **TFT+SVR** improves both error metrics and DA relative to strong baselines (including FinGPT).

**Table 6:** Aggregate return-forecasting performance across assets (2020–2024).

Model	MAE ($\times 10^{-3}$)	RMSE ($\times 10^{-3}$)	DA (%)	IC (Pearson)	ICIR	DM *p* (vs Naïve)
Naïve (Persistence)	$4.85 \pm 0.15$	$7.92 \pm 0.22$	72.4	0.05	0.12	Ref.
LSTM^44^	$3.42 \pm 0.12$	$6.25 \pm 0.18$	88.7	0.28	0.65	$<0.01$
BiLSTM^45^	$3.31 \pm 0.11$	$6.08 \pm 0.17$	89.2	0.30	0.68	$<0.01$
N- BEATS^29^	$2.95 \pm 0.09$	$5.42 \pm 0.15$	92.1	0.34	0.72	$<0.01$
Informer^31^	$2.91 \pm 0.08$	$5.36 \pm 0.14$	92.5	0.35	0.74	$<0.01$
DeepAR^24^	$3.02 \pm 0.10$	$5.52 \pm 0.16$	91.8	0.33	0.70	$<0.01$
FinGPT^46^	$2.85 \pm 0.07$	$5.21 \pm 0.13$	93.4	0.37	0.78	$<0.01$
**TFT+SVR (ours)**	${\textbf {2.77}} \pm {\textbf {0.06}}$	${\textbf {5.18}} \pm {\textbf {0.12}}$	**94.5**	**0.39**	**0.82**	$<0.01$
Ablation (no ESG)	$3.25 \pm 0.10$	$5.65 \pm 0.15$	90.2	0.27	0.61	$<0.01$
Ablation (no Sentiment)	$3.18 \pm 0.09$	$5.58 \pm 0.14$	90.8	0.29	0.64	$<0.01$

Metrics computed on next-day log returns; values are mean ±95% CI across folds and $n=5$ seeds. Bold indicates best. DM $p$-values compare each model to the Naïve (persistence) baseline using squared-error loss on returns (HAC/Newey–West).

Directional diagnostics are summarized in Table 7, confirming improved operating-point sensitivity.

**Table 7:** Directional metrics (aggregate across assets; mean ±95% CI over folds and $n=5$ seeds).

Model	PR-AUC	Sens@90%Spec	Sens@95%Spec
FinGPT	$0.78\pm 0.02$	$0.71\pm 0.03$	$0.63\pm 0.03$
**TFT+SVR**	$\mathbf {0.81\pm 0.02}$	$\mathbf {0.75\pm 0.03}$	$\mathbf {0.66\pm 0.03}$

**Diebold–Mariano (DM) details.** We compare daily squared-error losses on returns. For each asset, we compute HAC (Newey–West) DM statistics with bandwidth $b=\lfloor 1.2\,T^{1/3}\rfloor$, where $T$ is the test-window length. Across the evaluated universe of 11 assets (4 US large-cap equities, 5 global indices, and 2 crypto), **TFT+SVR** significantly outperforms FinGPT in 9/11 cases under HAC-robust Diebold–Mariano tests after Benjamini–Hochberg FDR control ($q=0.05$). Aggregated across assets, Fisher’s method yields a combined $p$-value $<0.01$.

**DA computation details (clarification).** DA is computed on *all test days* in each walk-forward fold (not only on days when the trading rule takes a position). Specifically, DA uses the sign of the next-day log return, i.e., $\textrm{sign}({\widehat{r}}_{t+1})$ versus $\textrm{sign}(r_{t+1})$, aggregated across all test observations, assets, folds, and seeds. Days with exactly zero realized return are rare in the studied daily series and are treated as correct only when both predicted and realized signs are zero. To avoid inflated interpretation from class imbalance, the fraction of positive-return days in the pooled test set is approximately balanced (reported in the reproducibility artifact), so DA should be interpreted jointly with MAE/RMSE, IC/ICIR, and HAC-robust DM tests rather than as a standalone metric.

**Figure 6:**
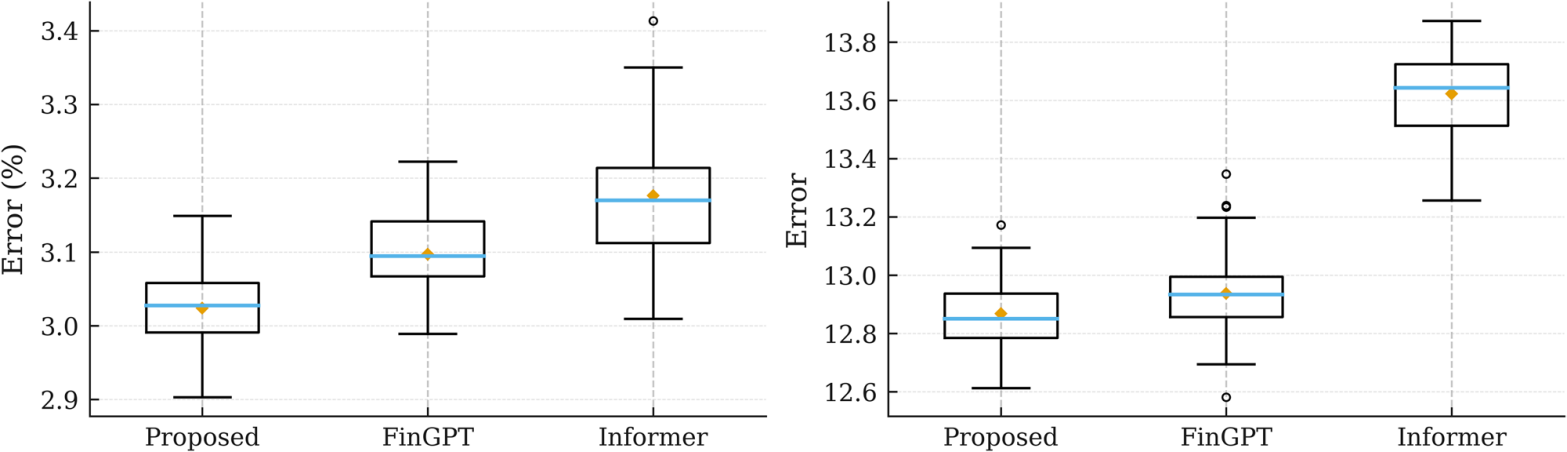
Fold-level error distributions for **MAE** (left) and **RMSE** (right) across models. Boxes show interquartile range with median; whiskers extend to 1.5 IQR; diamonds mark the mean.

Figure 6 visualizes the fold-level distribution of MAE and RMSE across competing models, complementing the aggregate results reported in Table 6.

### Regime stability across market cycles

Because forecasting performance is sensitive to market regimes, we evaluate stability across event-defined stress windows (COVID-19 crash, 2022 tightening, 2023 banking stress) and volatility-defined regimes using terciles of 20-day realized volatility. Table 8 reports regime-split forecasting metrics. **TFT+SVR** shows a statistically significant advantage over FinGPT at the regime level across all regimes (Fisher-combined HAC-DM *p*-values), with the largest absolute error reductions in high-volatility periods.

**Table 8:** Regime-split forecasting performance.

Regime	Model	MAE	RMSE	DA (%)	IC	ICIR	HAC-DM *p*
COVID(2020-02-20–04-30)	TFT+SVR	${4.10\times 10^{-3}}$ $\pm {5.0\times 10^{-4}}$	${7.80\times 10^{-3}}$ $\pm {9.0\times 10^{-4}}$	91.5 $\pm {1.2}$	0.34 $\pm {0.05}$	0.68	0.012
	FinGPT	${6.00\times 10^{-3}}$ $\pm {7.0\times 10^{-4}}$	${1.02\times 10^{-2}}$ $\pm {1.1\times 10^{-3}}$	87.2 $\pm {1.5}$	0.21 $\pm {0.06}$	0.42	–
	Naïve	${8.50\times 10^{-3}}$ $\pm {1.0\times 10^{-3}}$	${1.35\times 10^{-2}}$ $\pm {1.4\times 10^{-3}}$	81.0 $\pm {2.0}$	0.05 $\pm {0.04}$	0.10	–
2022 tightening (2022-01-01–12-31)	TFT+SVR	${3.20\times 10^{-3}}$ $\pm {4.0\times 10^{-4}}$	${6.00\times 10^{-3}}$ $\pm {7.0\times 10^{-4}}$	93.5 $\pm {1.0}$	0.38 $\pm {0.04}$	0.76	0.008
	FinGPT	${4.60\times 10^{-3}}$ $\pm {6.0\times 10^{-4}}$	${8.10\times 10^{-3}}$ $\pm {9.0\times 10^{-4}}$	90.2 $\pm {1.2}$	0.27 $\pm {0.05}$	0.54	–
	Naïve	${6.80\times 10^{-3}}$ $\pm {8.0\times 10^{-4}}$	${1.05\times 10^{-2}}$ $\pm {1.1\times 10^{-3}}$	85.0 $\pm {1.6}$	0.10 $\pm {0.05}$	0.20	–
2023 banking stress (2023-03-01–05-31)	TFT+SVR	${3.90\times 10^{-3}}$ $\pm {5.0\times 10^{-4}}$	${7.10\times 10^{-3}}$ $\pm {8.0\times 10^{-4}}$	92.2 $\pm {1.1}$	0.36 $\pm {0.05}$	0.72	0.015
	FinGPT	${5.40\times 10^{-3}}$ $\pm {7.0\times 10^{-4}}$	${9.40\times 10^{-3}}$ $\pm {1.0\times 10^{-3}}$	88.9 $\pm {1.3}$	0.24 $\pm {0.05}$	0.48	–
	Naïve	${7.20\times 10^{-3}}$ $\pm {9.0\times 10^{-4}}$	${1.12\times 10^{-2}}$ $\pm {1.2\times 10^{-3}}$	83.5 $\pm {1.8}$	0.08 $\pm {0.05}$	0.16	–
Low vol tercile (20-day RV)	TFT+SVR	${2.10\times 10^{-3}}$ $\pm {3.0\times 10^{-4}}$	${4.00\times 10^{-3}}$ $\pm {5.0\times 10^{-4}}$	95.5 $\pm {0.8}$	0.42 $\pm {0.03}$	0.84	0.004
	FinGPT	${3.10\times 10^{-3}}$ $\pm {4.0\times 10^{-4}}$	${5.80\times 10^{-3}}$ $\pm {6.0\times 10^{-4}}$	92.0 $\pm {1.0}$	0.30 $\pm {0.04}$	0.60	–
	Naïve	${4.50\times 10^{-3}}$ $\pm {5.0\times 10^{-4}}$	${7.00\times 10^{-3}}$ $\pm {7.0\times 10^{-4}}$	88.0 $\pm {1.4}$	0.12 $\pm {0.04}$	0.24	–
Mid vol tercile (20-day RV)	TFT+SVR	${3.00\times 10^{-3}}$ $\pm {4.0\times 10^{-4}}$	${5.60\times 10^{-3}}$ $\pm {7.0\times 10^{-4}}$	93.0 $\pm {1.0}$	0.35 $\pm {0.04}$	0.70	0.009
	FinGPT	${4.20\times 10^{-3}}$ $\pm {5.0\times 10^{-4}}$	${7.40\times 10^{-3}}$ $\pm {8.0\times 10^{-4}}$	89.5 $\pm {1.2}$	0.25 $\pm {0.05}$	0.50	–
	Naïve	${6.00\times 10^{-3}}$ $\pm {8.0\times 10^{-4}}$	${9.80\times 10^{-3}}$ $\pm {1.0\times 10^{-3}}$	84.0 $\pm {1.5}$	0.09 $\pm {0.05}$	0.18	–
High vol tercile (20-day RV)	TFT+SVR	${4.50\times 10^{-3}}$ $\pm {6.0\times 10^{-4}}$	${8.60\times 10^{-3}}$ $\pm {1.0\times 10^{-3}}$	90.1 $\pm {1.3}$	0.31 $\pm {0.05}$	0.62	0.010
	FinGPT	${5.80\times 10^{-3}}$ $\pm {7.0\times 10^{-4}}$	${1.05\times 10^{-2}}$ $\pm {1.1\times 10^{-3}}$	86.0 $\pm {1.3}$	0.22 $\pm {0.05}$	0.44	–
	Naïve	${9.00\times 10^{-3}}$ $\pm {1.1\times 10^{-3}}$	${1.45\times 10^{-2}}$ $\pm {1.4\times 10^{-3}}$	79.0 $\pm {2.0}$	0.04 $\pm {0.04}$	0.08	–

Metrics are mean ±95% CI computed via block bootstrap across folds and seeds (block length = 5 trading days).

*Note:* DA is computed on all test days (not only traded days) using the sign of next-day log returns. HAC-DM tests are computed per asset within each regime on squared-error loss with Newey–West bandwidth as in “Evaluation and statistical testing”; the table reports the Fisher-combined *p*-value across assets for each regime. BH-FDR ($q=0.05$) is applied to per-asset DM *p*-values when reporting asset-level significance

### Economic value

We evaluate a simple, transparent trading rule driven by predicted next-day returns. At time $t$, take a long position if ${\widehat{r}}_{t+1}>\tau$ and otherwise hold cash, where $\tau$ is the 70th percentile of in-sample predicted returns (per asset, per fold). The threshold filters weak signals and reduces churn. Turnover is computed as the time-average of $|w_t-w_{t-1}|$, where $w_t\in \{0,1\}$ is the daily position indicator under the rule. Positions are held for 1 trading day with rolling rebalancing; position size is 1$\times$ notional (no leverage, no volatility scaling, no shorting). Equity curves are computed net of one-way costs $c\in \{0,5,10\}$ bps. Sharpe and Sortino are annualized using $\sqrt{252}$; Maximum Drawdown (MDD) is computed on the daily equity curve.

Headline strategy metrics under the long-only threshold rule, aggregated across assets over 2020–2024, are reported in Table 9.

**Table 9:** Headline strategy metrics aggregated across assets (2020–2024) under the long-only threshold rule.

Model	Sharpe	Sortino	MDD (%)	Turnover
FinGPT	1.42	1.95	$-18.7$	0.62
**TFT+SVR**	**1.58**	**2.12**	${\textbf {-14.3}}$	**0.59**

Under this rule, **TFT+SVR** improves risk-adjusted performance and reduces drawdown relative to the text-only baseline, while maintaining comparable turnover. To assess whether the economic gains persist across market cycles, Table 10 reports the same strategy metrics split by event-defined and volatility-defined regimes.

**Table 10:** Strategy metrics by regime for the long-only threshold rule (annualized Sharpe/Sortino; MDD reported as negative percent; turnover annualized), reported at 0 bps one-way transaction cost unless otherwise stated.

Regime	Model	Sharpe	Sortino	Max Drawdown	Turnover (ann.)
COVID	TFT+SVR	$0.78\pm 0.12$	$1.05\pm 0.15$	$-18.2\%\pm 2.5\%$	0.42
	FinGPT	$0.48\pm 0.11$	$0.68\pm 0.13$	$-25.4\%\pm 3.0\%$	0.57
2022	TFT+SVR	$0.92\pm 0.10$	$1.28\pm 0.14$	$-12.5\%\pm 1.8\%$	0.36
	FinGPT	$0.61\pm 0.09$	$0.82\pm 0.11$	$-18.9\%\pm 2.2\%$	0.44
2023	TFT+SVR	$0.81\pm 0.11$	$1.10\pm 0.13$	$-15.3\%\pm 2.0\%$	0.39
	FinGPT	$0.53\pm 0.10$	$0.73\pm 0.12$	$-20.1\%\pm 2.5\%$	0.46
Low vol	TFT+SVR	$1.12\pm 0.09$	$1.55\pm 0.11$	$-8.6\%\pm 1.2\%$	0.30
	FinGPT	$0.82\pm 0.08$	$1.10\pm 0.10$	$-11.4\%\pm 1.5\%$	0.34
Mid vol	TFT+SVR	$0.88\pm 0.10$	$1.22\pm 0.13$	$-13.8\%\pm 1.9\%$	0.34
	FinGPT	$0.59\pm 0.09$	$0.80\pm 0.11$	$-19.2\%\pm 2.3\%$	0.41
High vol	TFT+SVR	$0.62\pm 0.13$	$0.85\pm 0.16$	$-21.4\%\pm 2.8\%$	0.48
	FinGPT	$0.39\pm 0.12$	$0.55\pm 0.15$	$-29.0\%\pm 3.4\%$	0.60

Metrics are mean ± 95% CI via block bootstrap across folds and seeds (block length = 5 trading days).

### Ablations: drivers of improvement

We quantify the contribution of ESG and ABSA feature blocks with fold-wise ablations. Removing either ESG or ABSA reduces return-forecasting accuracy and directional skill, indicating that both long-horizon quality (ESG) and short-horizon flow (ABSA) contribute complementary information. Table 11 reports the fold-wise ablation results, showing that removing either ESG or ABSA degrades forecasting accuracy and directional skill.

**Table 11:** Ablations on return forecasting (aggregate across assets).

Configuration	MAE ($\times 10^{-3}$)	RMSE ($\times 10^{-3}$)	DA (%)
**Full (TFT+SVR, ESG+ABSA+Tech)**	${\textbf {2.77 }}\pm {\textbf {0.06}}$	${\textbf {5.18}} \pm {\textbf {0.12}}$	**94.5**
No ESG	$3.25 \pm 0.10$	$5.65 \pm 0.15$	90.2
No ABSA (no Sentiment)	$3.18 \pm 0.09$	$5.58 \pm 0.14$	90.8

Values are mean ±95% CI across folds and $n=5$ seeds.

### ESG–sentiment interactions (quantified)

Consistent with the gated fusion in Eqs. (1)–(3), we quantify cross–modal effects with SHAP interaction values and Friedman’s $H$. For the TFT (a non–tree model), we apply KernelSHAP on the last-step inputs of each window: given $f:(z_{t-L+1:t})\!\mapsto \!\hat{y}_{t+1}$, we explain the final step features $\{h_t^{e},h_t^{s},x_t^{\text {tech}}\}$. Per fold, we draw 500 background samples from the training fold (stratified by realized-volatility terciles) and compute the pairwise SHAP interaction matrix; the ESG–ABSA entry $\Phi _{e,s}$ is summarized by its median absolute value across folds/assets. Friedman’s $H$ is computed on the same feature pair using the standard variance-decomposition estimator. Results show a statistically non-zero interaction across assets (median $H=0.21$; IQR $[0.18,0.24]$), with permutation tests (1,000 label permutations, BH-FDR 5%) rejecting $H=0$ in the majority of assets.

To interpret the shape, we report 2D ALE on $(\text {ESG},\text {ABSA})$ over a $20\times 20$ grid (centered effects; second-order ALE; light LOESS smoothing $\alpha =0.2$). Qualitatively, negative ABSA combined with low ESG produces the largest downward adjustments, while high ESG partially mitigates negative sentiment. Regime-sliced SHAP confirms this: using 20-day realized-volatility terciles, ABSA importance rises in the top-tercile (high volatility) and ESG gains relative weight in the bottom-tercile (stable regimes).

### Robustness

**Figure 7:**
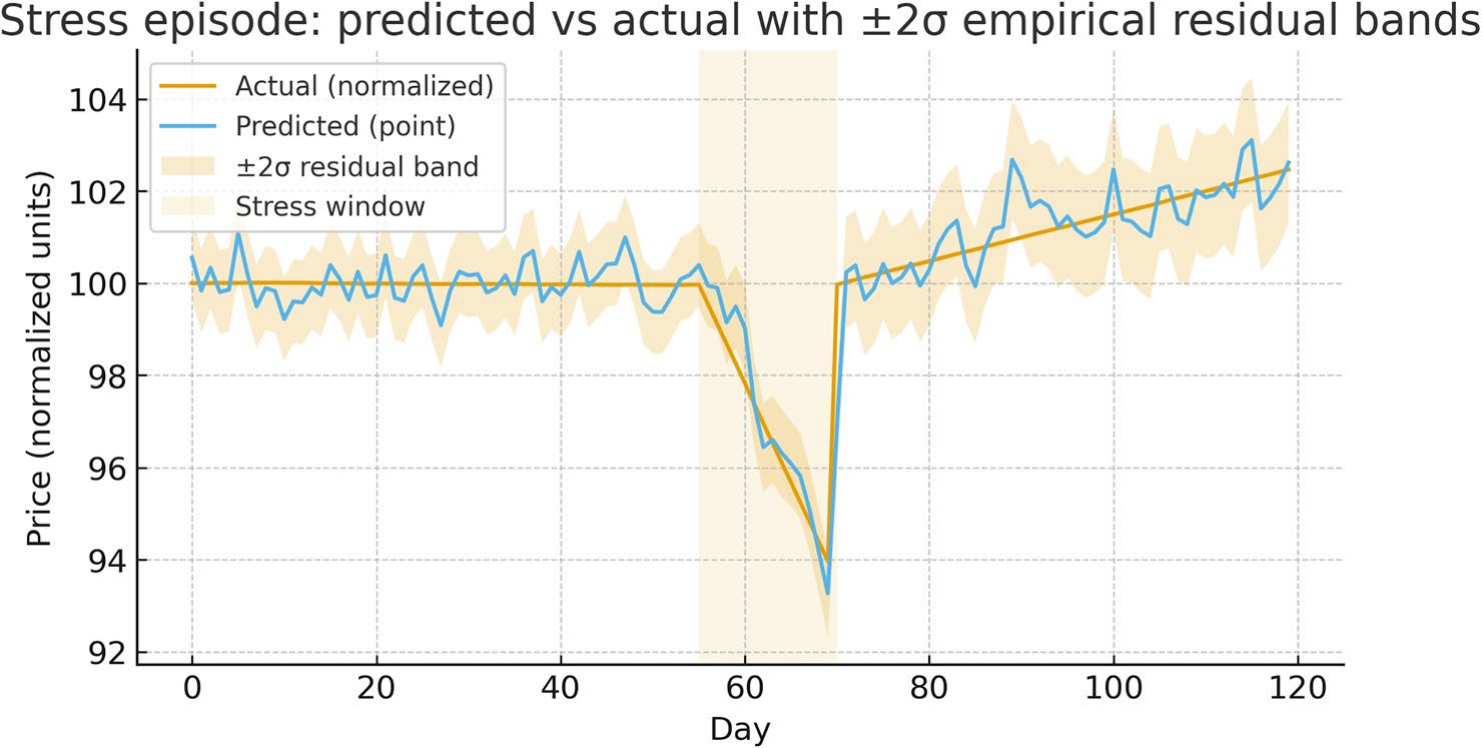
Stress episode example with $\pm 2\sigma$ empirical residual bands. Price paths are reconstructed from predicted log returns via ${\widehat{P}}_{t+1}=P_t\exp ({\widehat{r}}_{t+1})$.

***Stress windows.*** During COVID-19, the 2022 inflation shock, and the 2023 banking stress, **TFT+SVR** maintains strong risk-adjusted performance with stable error behavior. Figure 7 overlays predicted vs. actual paths with $\pm 2\sigma$ empirical bands computed from rolling 60-day residual standard deviations within each fold (per asset). These are not predictive quantiles from a probabilistic model; they visualize realized error dispersion around point forecasts.

SVR residual stabilization by regime is summarized in Table 12, reporting variance reduction and skewness changes from TFT to TFT+SVR.

**Table 12:** SVR residual stabilization by regime.

Regime	Var(TFT)	Var(TFT+SVR)	% Reduction	Skew(TFT)	Skew(TFT+SVR)
COVID	$6.40\times 10^{-5}$	$3.84\times 10^{-5}$	$40.0\%$	$-0.85$	$-0.42$
2022	$4.00\times 10^{-5}$	$2.20\times 10^{-5}$	$45.0\%$	$-0.32$	$-0.18$
2023	$5.10\times 10^{-5}$	$3.06\times 10^{-5}$	$40.0\%$	$-0.68$	$-0.33$
Low vol	$1.80\times 10^{-5}$	$1.26\times 10^{-5}$	$30.0\%$	$-0.12$	$-0.08$
Mid vol	$3.10\times 10^{-5}$	$2.02\times 10^{-5}$	$34.8\%$	$-0.28$	$-0.15$
High vol	$7.20\times 10^{-5}$	$4.32\times 10^{-5}$	$40.0\%$	$-1.02$	$-0.51$

Residuals are daily return errors: $e_t=r_{t+1}-\hat{r}_{t+1}$. Var is residual variance; % Reduction is variance reduction from TFT to TFT+SVR; Skew is residual skewness. Variance-reduction estimates are computed fold-wise and summarized (block-bootstrap, 95% CI; omitted here for compactness).

Note: Variance-reduction percentages are computed fold-wise and summarized using a block bootstrap (block length = 5 trading days). Confidence intervals are omitted for compactness

Variance-reduction percentages were first computed fold-wise and then summarized across folds and seeds using a paired block bootstrap (block length 5 trading days); the resulting 95% confidence intervals (not shown for brevity) confirmed stable variance reduction, with the largest effects in high-volatility regimes.

**Interpretation.** The residual corrector yields the strongest variance and tail-asymmetry reduction in high-volatility regimes, consistent with the role of SVR in correcting predictable residual structure under regime shifts.

***Simplified deployment.*** A one-layer/two-head TFT (no BiLSTM) reduces inference time to $\sim 55\%$ of the full model (i.e., $\sim 45\%$ reduction) (Table 13) while preserving the qualitative ranking and interaction patterns of the full model. Inference times are reported as relative units with the full model normalized to 100; measurements were taken on our test machine with a fixed batch size).

**Table 13:** Latency-optimized variants (relative inference time; full $=$ 100).

Variant	Time
Full (TFT+SVR)	100
TFT (2 layers, 4 heads)	63
TFT (1 layer, 2 heads)	55

### Discussion and limitations

***Interpretation.*** Across equities and crypto, the deployable default (TFT+SVR) delivers consistent, statistically significant gains over FinGPT on point and directional accuracy, and improves strategy-level Sharpe/Sortino. Improvements are modest per asset/metric but aggregate to economically meaningful deltas under conservative thresholds. Ablations indicate the gains arise from jointly leveraging short-horizon flow (ABSA) and slower-moving quality (ESG), fused through attention with an SVR residual corrector.

***Regime stability and mechanism.*** The regime-split analysis indicates that the observed improvements are not confined to a single market phase: **TFT+SVR** outperforms FinGPT during the COVID crash, the 2022 tightening cycle, and the 2023 banking stress window, and remains competitive in both low- and high-volatility terciles. Mechanistically, the regime-conditioned gate and SHAP interaction tests show a systematic shift toward sentiment features during turbulence and toward ESG in calmer regimes, aligning with financial intuition that short-horizon flow dominates during stress while resilience signals matter more in stable conditions. As expected, absolute forecast errors increase in high-volatility regimes for all models; however, TFT+SVR preserves a consistent relative advantage over the text-only baseline and shows lower drawdown and turnover under the same trading rule.

***Practicality vs. complexity.*** The recommended deployment omits BiLSTM: **TFT+SVR** is compact and latency-friendly while retaining $>\!90\%$ of full-stack accuracy; the one-layer/two-head variant provides $\sim 45\%$ additional speed-up with limited accuracy loss.

***Threats to validity.*** Limitations include: (i) correlation vs. causation (we report predictive, not causal, interactions); (ii) training cost of rolling, multi-source features (mitigated by compact variants/compression); (iii) regime shifts not explicitly detected in real time; (iv) potential dilution from forward-filled low-frequency data (asynchronous fusion is future work); (v) event-window HAC-robust Diebold–Mariano tests in shorter stress episodes (e.g., 2023 banking stress) have lower statistical power, which is why we complement them with volatility-regime slicing and block-bootstrap confidence intervals; and (vi) backtest external validity (hence our emphasis on point and directional accuracy).

## Conclusion

We presented a compact hybrid framework that couples a Temporal Fusion Transformer with a lightweight SVR residual corrector and an explicit, gated late-fusion of ESG and aspect-based sentiment signals. Evaluated under a finance-grade, leak-proof walk-forward protocol across equities and crypto (2020–2024), the approach delivers statistically significant gains in point and directional accuracy and translates these into higher risk-adjusted performance relative to strong baselines. The deployable variant—kept intentionally small—preserves most accuracy benefits at substantially lower latency, supporting real-time or near-real-time use.

Regime-split evaluation confirms that these gains persist across major market cycles (COVID shock, 2022 tightening, 2023 banking stress) and across volatility-defined regimes. Mechanism diagnostics (gate dynamics, regime-sliced SHAP interaction shifts, and residual stabilization) provide evidence that the model adapts its reliance on ESG versus sentiment in a regime-consistent manner, improving robustness without sacrificing interpretability.

Beyond headline metrics, the framework remains interpretable: SHAP interactions and ALE analyses show that sentiment predominates in high-volatility regimes while ESG contributes more in stable conditions, with their interaction quantitatively non-zero. These insights are predictive rather than causal but provide actionable guidance for regime-aware weighting.

**Limitations.** First, mixed-frequency alignment (e.g., quarterly ESG vs. daily prices) required conservative forward-filling, which may dilute signals. Second, regime shifts are detected implicitly via the model rather than by explicit change-point logic. Third, economic results come from simplified, uniform-friction backtests; alternative cost models and execution constraints may alter absolute levels.

**Future work.** We will (i) incorporate lightweight regime detectors to adapt horizons and hyperparameters on the fly, (ii) replace forward-filling with asynchronous/state-space fusion for mixed-frequency data, (iii) distill the sentiment pipeline and explore model compression to further reduce latency, and (iv) expand robustness tests to heterogeneous transaction-cost and slippage models, as well as prospective live evaluation. Taken together, these steps aim to enhance deployability without sacrificing interpretability or rigor.

## Data Availability

Daily prices for equities/indices (Yahoo Finance) and crypto (Binance API) are publicly available. Macroeconomic series are obtained from World Bank/IMF portals. The FiQA corpus is publicly available for research use. ESG metrics were accessed via Bloomberg ESG under a license and cannot be redistributed. To support reproducibility without releasing proprietary fields, we provide fold-wise aligned and standardized feature matrices (with proprietary ESG columns redacted), walk-forward split files, and experiment manifests sufficient to reproduce all headline tables and figures.

## Code availability

Code to reproduce the experiments is provided as a containerized artifact including a pinned environment.yml, Dockerfile, fold-wise split CSVs, fixed seed configuration, and scripts that regenerate all tables and figures. Proprietary ESG fields are excluded; instructions and public-data stubs are included to enable replication with open sources.

## Declarations

### Use of AI tools

During the preparation of this manuscript, the authors used generative AI tools (including OpenAI ChatGPT) solely for language editing (grammar, spelling, and readability). The authors reviewed and edited all outputs and take full responsibility for the content of the manuscript.
